# Effect of simultaneous physical and auditory stressors on cardiorespiratory response

**DOI:** 10.1038/s41598-025-96845-3

**Published:** 2025-04-15

**Authors:** Christian Buj, Meret Preuß, Maximilian Mörsdorf, Anke Schmidt, Denise Guckel, Daniel Dumitrescu, Franziska Klein, Lisa Straetmans-Oehme, Marco Eichelberg, Andreas Hein

**Affiliations:** 1 R&D Division Health, OFFIS-Institute for Information Technology, Oldenburg, Germany; 2 Herz- und Diabeteszentrum NRW, Universitätsklinikum der Ruhr-Universität Bochum, Bad Oeynhausen, Germany; 3 Department of Psychology, University of Oldenburg, Oldenburg, Germany; 4 Department of Assistance Systems and Medical Device Technology, University of Oldenburg, Oldenburg, Germany

**Keywords:** Occupational health, Biomedical engineering

## Abstract

In occupational medicine, monitoring individual stress-related physiological responses is an effective tool for minimizing health risks at the workplace. From an audiology perspective, this particularly concerns the effects of auditory stress, which leads to increased listening effort with subsequent hearing fatigue. A study was conducted to investigate whether cardio-respiratory responses can detect the effects of a multi-level combination of physical and auditory stressors. To investigate their measurability and determine whether an interaction exists, a selection of cardio-respiratory vital parameters such as heart rate, features in the time and frequency domain of the heart rate variability, breathing rate, respiratory minute volume, and the respiratory quotient were analyzed. The results showed a significant main effect of physical stress on all assessed parameters. Auditory stress demonstrated a significant impact on breathing frequency, root mean square of successive differences of interbeat intervals, and the power components of the low and high frequency bands of the heart rate variability. No interaction between auditory and physical stressors was observed across any of the examined parameters. From these results we conclude that physiological responses to different sources of stress can be recorded within selected vital parameters, independent of external stimuli such as ambient noise. In an occupational context, we see potential in tracking individual auditory stress by monitoring cardio-respiratory parameters, especially breathing patterns. By knowing the individual (auditory) stress level, conclusions could be drawn about the worker’s ability to concentrate and further measures could be taken to combat safety risks in the work environment.

## Introduction

As a broad topic in the field of application and research of audiology, determining the individual level of auditory stress can provide tools for reducing listening effort and thus reducing hearing fatigue^1–3^. Particularly in the context of occupational medicine, by monitoring individual auditory stress levels and vital parameters, health risks could be minimized^4^. For instance, Saha et al. were able to show that noise in a work environment led to a significant increase in heart rate and blood pressure in workers working in a thermal power plant^5^. Persistently elevated heart rate and arterial hypertension represent significant risk factors for cardiovascular diseases, including heart attacks and strokes. Chronic stress and occupational stressors may further exacerbate these pathophysiological conditions^6^. Furthermore, chronic auditory stress has been found to influence the circadian rhythm changes in heart rate variability [ms] (HRV)^7^.

The term *stress* is defined differently in the literature, usually depending on the context of the respective work^8^. Here we define stress as the result of a stimulus that creates a non-homeostatic situation for the human body and therefore places greater demands on cognitive and/or physical performance. With this we refer to the contextualization of Everly et. al.^9^. There, stress is defined as *a physiological reaction, or response, regardless of the source of the reaction* and therefore corresponds to Hans Selye’s frequently used definition of stress: *Stress is the nonspecific response of the body to any demand made upon it*^10^. In addition, we are adjusting the use of the term *stressor* as a *stimulus that evokes a stress response* like physical exercises (stress), as well as the categorization into psychosocial and biogenic stressors. The former arises from a cognitive interpretation of an event, such as an auditory stimulus. Biogenic stressors do not require interpretation, but are created by a direct reaction, for example, to substances such as tea, coffee or physical exercise^9^. Latter have a direct effect on the body such as metabolic or physiological changes^11^.

Neurophysiological methods such as electroencephalography (EEG)^12,13^ or pupil dilation^14,15^ have proven to be particularly effective in detecting listening effort as they can measure the related effects. However, in most work-related situations, people are exposed to other potentially stressful stimuli in addition to auditory stress - in most cases it is some kind of biogenic stressor such as high physical workload. Physical stress is often associated with motion artifacts, making recording skin surface potentials with the sensitivity required for EEG recordings a major challenge. For this reason, the extraction of additional features from vital sign monitoring are chosen in the present study to determine the auditory stress level, which are briefly presented and motivated in the following. The HRV represents the variation in time intervals between consecutive heartbeats called interbeat intervals (s) (IBIs). These and the heart rate ($\textrm{min}^{-1}$) (HR) are constantly changing, allowing the cardiovascular system to adapt to the requirements of physical and psychological challenges^16,17^. As a central component of the stress response^18^, the HRV results from both heart-brain interactions and dynamic non-linear processes in the autonomic nervous system^17^. Anatomically and functionally, the autonomic nervous system (ANS) can be divided into the areas of the sympathetic nervous system (SNS), parasympathetic nervous system (PNS), and enteric nervous system (ENS). While ENS controls digestion within the gastrointestinal tract, SNS and PNS are complex antagonists in the (unconscious) regulation of organ function and homeostasis. In general, the SNS triggers ergotropic (activity-enhancing / “fight or flight”) responses in contrast to the trophotropic (relaxation-enhancing / “rest and digest”) processes of the PNS^19^.

In addition, and as shown in^20^, breathing patterns also change in response to applied stress. The volume of air that is individually ventilated per minute, also known as respiratory minute volume ($\textrm{min}^{-1}$) (VE), can be simultaneously regulated by the depth of each breath, known as tidal volume (L) (VT), and by the breathing frequency ($\textrm{min}^{-1}$) (BF). While the changes in VE during physical exercise are precisely regulated by hydrogen ion concentration in the arterial blood^21^, the dynamic regulation of breathing patterns is a complex physiological process that is not yet completely understood. During physical exercise, VE is primarily increased via a higher tidal volume at lower work intensity, while an additional increase in breathing frequency is only observed at higher work intensity^20^. The respiratory parameters can be quantified using a spirometer, for example, which measures respiration by detecting airflow as a person breathes into a mouthpiece, using sensors or mechanical systems to calculate air volume and flow rate.

Due to the relevance of stress for occupational health and the measurement difficulties, we conducted an exploratory study with the hypothesis stating that - in the presence of both auditory and physical multi intensity level stressors - effects of both can be detected in the analysis of cardio-respiratory vital features. We assume an increase of the cardio-respiratory response by an increase of the stimulation intensity in combination with an additive interaction between physical and auditory stimuli. Given our hypothesis, a selection of both HRV-related metrics of the cardiac system and features of the respiratory system, is investigated in the scope of this study. It is also limited to young and healthy people, as the possible variations due to, for example, existing diseases and/or the use of medication or drugs makes it impossible to interpret the causes or mechanisms.

## Materials and methods

### Prospective study

#### Participants

In order to recognize multilevel-stress responses, a prospective, randomized single-center study was carried out at the Heart- and Diabetes Center (Herz- und Diabeteszentrum NRW), Ruhr-Universität Bochum, Bad Oeynhausen (Germany) on 21 healthy adults. The study was reviewed and approved by the ethics committee of the medical faculty of the Ruhr University Bochum. Due to technical problems during recording, three participants were excluded. The remaining participants were on average (± SD) $\approx 26 \pm 6$ years (9 female and 9 male).

#### Study design

For the study, only healthy participants between the ages of 18 and 60 were selected. In addition, pregnant and breastfeeding women and people with a known allergy to propylene glycol (adhesive of the electrocardiogram (ECG) electrodes) were also excluded. The participants were paid an expense allowance of 50 €.

Before the study was carried out, all participants underwent an informed consent discussion and confirmed this in a written declaration of consent. Afterwards, a general questionnaire was completed to ascertain concomitant diseases and factors that could influence the outcome of the study.

During the study, participants were exposed to different combinations of physical and auditory stress. Physical stress was achieved by walking on a treadmill with different levels on inclination. Auditory stress was induced by asking participants to focus on the content of a story in either a single- or dual-speaker scenario, which based on the competing speaker paradigm by Donald Broadbent^22^ to analyze the dichotic listening effects^23^. To account for habituation effects, a pseudo-randomized decision was made to decide which of the two scenarios a participant started with.

The auditory stimulus was provided via the Portable Hearing Laboratory (PHL)^24^. The sound level of the acoustic stimulus was adjusted to the noise level of the treadmill due to the operation with different loads. This adjustment was intended to ensure that participants could hear the auditory input with comparable loudness and clarity. Before the measurements, all study participants underwent a 6-minute walk distance (6MWD) test^25–27^. In order to achieve one comfortable and one strenuous individual physical stress level on the treadmill, the 6MWD enables a standardized calculation of the treadmill speed, depending on the physical condition of the participants. This created three levels of physical stress:

- physical stress level 0: participants are sitting on a chair.
- physical stress level 1: treadmill speed calculated by the 6MWD, 1.5% slope resulting in ${35.83 \pm 10.88}$W on average.
- physical stress level 2: treadmill speed calculated by the 6MWD, 6% slope, resulting in ${74.56 \pm 20.20}$W on average.

During the study procedure, cardio-respiratory data were collected using a spiroergometer/metabolic cart (Amedtec Ergostix) with integrated 12 channel ECG (Amedtec ECGPro), wired in a standard configuration with six Einthoven or Goldberger limb leads and six Wilson chest wall leads^28^, and a Zephyr chest belt (Medtronic).

**Fig. 1:**
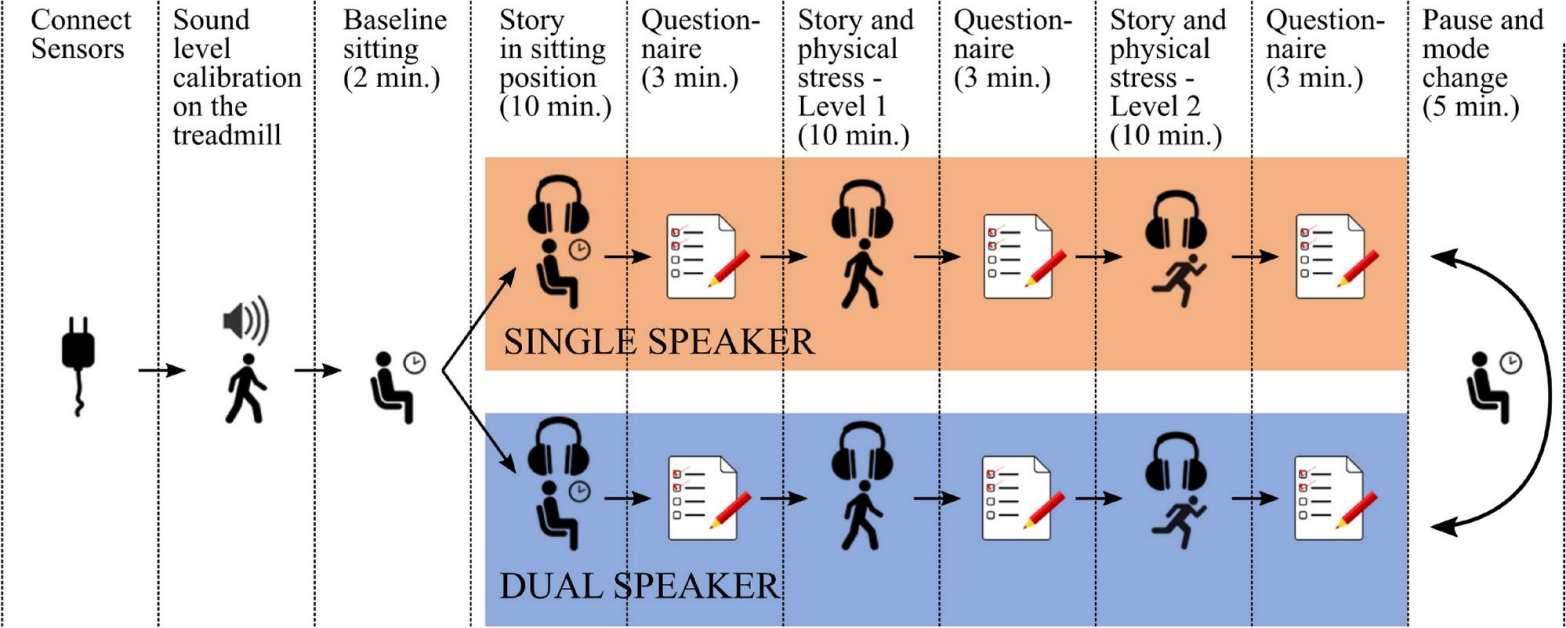
Overview of the structural procedure (study protocol) for data collection, which was performed in a study of 18 healthy participants.

As illustrated in Fig. 1, the experimental design consisted of two basic scenarios: the first is a scenario with a single speaker in which story A is told (single-speaker scenario, “s”). The second scenario is a two-speaker scenario where, in addition to story A heard in one ear, another story B heard in the other ear is read by second speaker (dual-speaker scenario, “d”). In both scenarios, there was a regular change in audio tracks between the right and left ears, while the study participants were asked to focus on story A. Both types of scenarios were repeated for each of the three physical stress levels (“0” for Level 0, “1” for “Level 1”, and “2” for “Level 2”). Combinations were labeled using a letter and number system to represent different conditions. For instance, codes s0 and d2 refer to conditions “single speaker audio + no physical stressor” and “dual speaker audio + level 2 physical stress”, respectively. The assignment of which scenario each participant starts with was pseudo-randomized to exclude order effects as far as possible. Within the scenarios, the order was always the same.

After each phase of physical stress, participants filled out a listening effort questionnaire (7-level rating from ”Effortless” - Score: 0 - to “Extremely Effortful” - Score: 6) and their perceived exertion due to fatigue and the content of the relevant story A (four multiple-choice questions)^29^. The categorical assessment levels of perceived listening effort were linked to ordinate values from 0 to 6 to allow for a comparative evaluation. The questions about the narrative content and perceived tiredness served to ensure the participants’ concentration and were, therefore, not further evaluated^30^.

The respective start and stop time points of the different study phases (cf. Fig. 1) were noted in a measurement protocol to enable phase-oriented data extraction as a necessary preprocessing step for the statistical analysis.

### Study systems and data processing

**Fig. 2:**
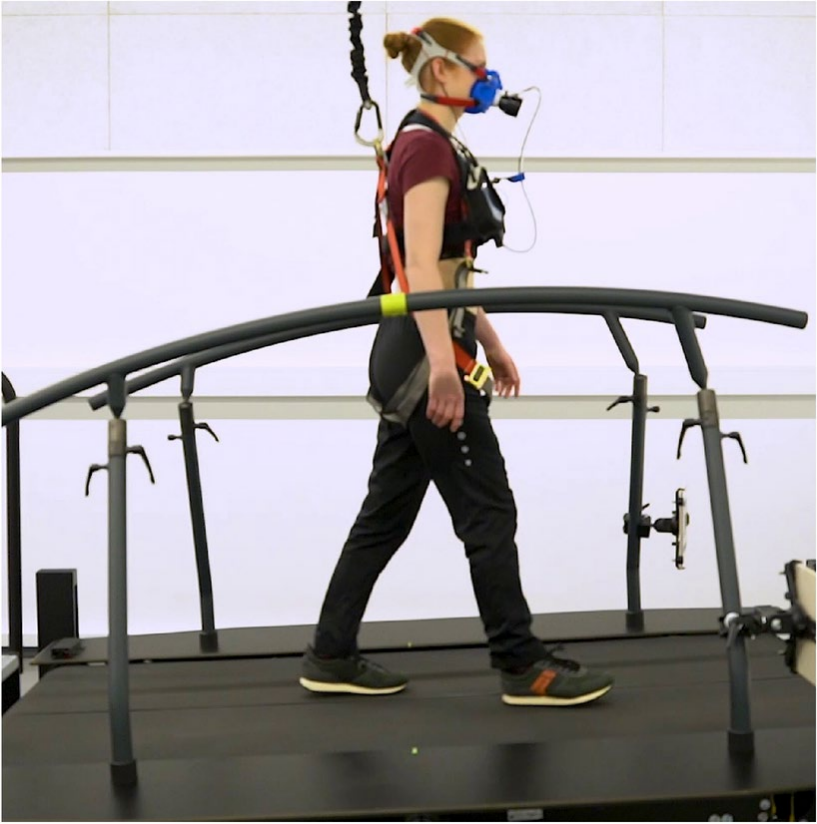
Experimental Setup: The participant is equipped with the spiroergometric system together with the Zephyr 1-lead ECG chest belt. Auditory stimuli are presented via binaural behind-the-ear hearing aids connected to the PHL. Physical stress is achieved using a running treadmill with two levels of inclination. To prevent injury due to potential tumbling or falling, the participant wore a harness.

To give an impression of the experimental setup, an exemplary data acquisition on the treadmill is shown in Fig. 2.

#### Sensor systems

The spiroergometric system is a combination of an ECG (Amedtec ECGPro CardioPart 12, 12-channel stress ECG), a spirometer (Amedtec Ergostik module S for measuring gas exchange) and a treadmill (h/p/cosmos quasar Med 4.0). The resulting measurements are generated through its export functions, resulting in an extensible markup language (XML) file comprising the ECG data and an OpenDocument Spreadsheet (ODS) file with a composite of the spirometer and treadmill data. The latter data are preprocessed by a moving average filter (window width 10 s), which corresponds to an effective sampling rate of 0.1 Hz. Due to synchronization problems of the different data acquisition systems, the individual study phases for each dataset had to be extracted manually using the study protocol and event markers in the ECG dataset. Additional plausibility tests, e.g., increase in heart rate during physical exertion, served as final confirmation. The short baseline sequences (2 min) were not accessible with sufficient certainty. In accordance with the finding that maintaining an ideally conditioned language requires minimal effort^31^, the experimental condition “s0” replaces the new baseline from now on.

Since strong motion artifacts were to be expected on the 12-lead ECG during the activity phases on the treadmill, a 1-lead ECG chest belt from Zephyr (BioModule device and Strap - Medtronic) was also used for measurement. The belt was connected to the PHL via lab streaming layer (LSL), which enables time-synchronized data recording^32^.

#### Spirometer and respiratory parameters

By analyzing of the spirometric data, the effects of auditory and physical stressors were investigated, which arise from the adaption of respiratory parameters. Due to the complexity of the adaptation described in the introduction, the parameters VE, BF, and the respiratory quotient (RQ) (quotient between carbon dioxide production and oxygen consumption) were analyzed.

#### ECG systems and cardiac parameters

ECG data were not extracted in raw format, but were filtered using a device-internal band-pass filter with cut-off frequencies of 0.5 Hz and 150 Hz as well as a notch filter with a cut-off frequency of 50 Hz. For the calculation of the ECG features, only the R peaks are of further interest. The R peak is the maximum amplitude in the R wave which describes the depolarization of the ventricles from the base to the apex of the heart^33^. Due to their greatest presence in the second Einthoven derivative due to the normal alignment of the heart’s electrical axis^34^, these data were considered.

To identify stress-dependent and measurable cardiac rhythm responses, the HR and the following HRV features were analyzed in the *time domain*:

- standard deviation of the NN intervall (SDNN): Specifies the average deviation from the mean normal-to-normal sinus beats interval (NN) and is measured in ms. “Normal” (cf.^35^) means that abnormal beats due to motion artifacts have been removed. However, since only healthy participants took part in the study and the HRV was calculated for the individual study sequences, this value corresponds to the standard deviation of all considered sinus beats (SDRR).
- root mean square of successive differences (RMSSD): Describes the beat-to-beat variance in HR and is a measure for estimating changes mediated by the vagus nerve, the main parasympathetic nerve^36^.

The following HRV parameters were taken into account in the *frequency domain* because they are highly correlated with the activity of the PNS and SNS^37^:

- Power of the low-frequency range between 0.04 Hz to 0.15 Hz (LF): In this frequency range, fluctuations are mainly influenced by the baroreceptor activity during resting conditions^38^. Acharya et al. have found that stress correlates with an increase in the power of Power of the low-frequency range between 0.04 Hz to 0.15 Hz (LF), which corresponds to an increase in sympathetic stimulation^39^.
- Power of the high-frequency range between 0.15 Hz to 0.4 Hz (HF): The The Power of the high-frequency range between 0.15 Hz to 0.4 Hz (HF)-band is generally associated with the activity of parasympathetic branch of the ANS. One cause of HR fluctuations is the breathing cycle- this process is known as respiratory sinus arrhythmia (RSA)^40^. Decreased HF power is associated with stress, panic, anxiety, or worry^17^.
- LF/HF ratio - Estimation of the ratio between SNS and PNS activity. When low, it reflects a dominance of PNS and vice versa for SNS activity.

#### Stimulus systems

Two stimulus systems were available for the study. One is represented by the treadmill from the spiroergometer system for generating physical stress and the other is the Portable Hearing Laboratory (PHL) to generate auditory stress. The PHL device is a wearable hearing aid research platform developed as part of the “Open community platform for hearing aid algorithm research” project. It consists of a Beaglebone Black single-board computer that has been expanded with a multi-channel audio interface “Cape4all”, a multi-channel audio board that is available under an open-source license. In addition, the functionality is provided by the open-source real-time audio signal processing platform “openMHA”^24^.

During the study, the stories were played to participants via the PHL’s binaural behind-the-ear hearing aids. The length of each study phase depended mainly on the length of the story, which varied slightly around 10min ($\pm {15}s$). Relevant information, such as the start and stop time stamps of playback sequences, was stored in a log file. These were used as additional information for the manual extraction of the study phases described above. In order to detect both initial and delayed changes, the analysis was initiated directly at the start of each phase and the maximum possible data length was used.

#### Data processing

**Fig. 3:**
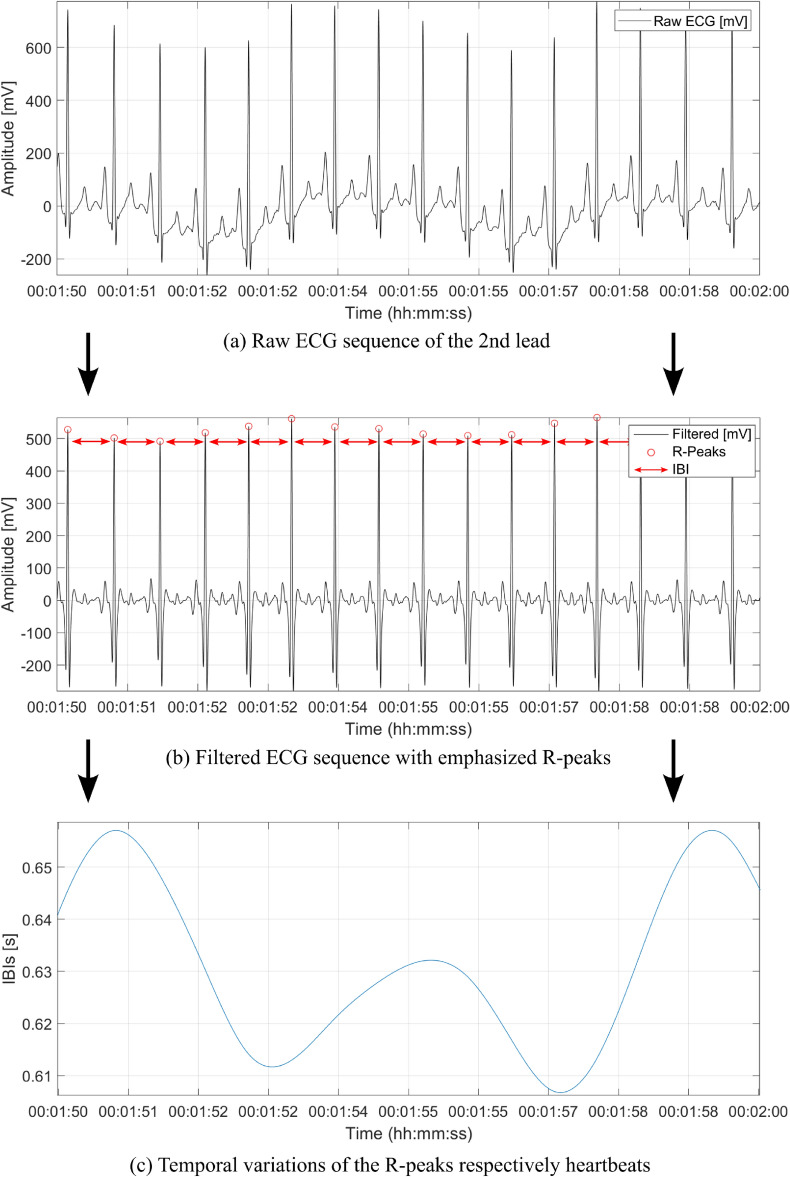
Exemplary illustration of the processing steps from the raw ECG signal to the heart rate variation of a 2nd channel to Einthoven of a single subject. (**a**) Illustrates a raw signal sequence of the ECG-signal. (**b**) The signal as processed by a wavelet filter, in which the R peaks are determined and highlighted by the red circle markers. (**c**) Visualisation of the heart rate variability as the variations of the interbeat intervals (IBIs), which are indicated in (**b**) by the red arrows.

Evaluation of the ECG data was carried out with MATLAB (version R2021b)^41^ and a specially tailored self-made graphical user interface (GUI) in which selected channels can be visualized, filtered and features extracted. All derived features are based on the HR or R peaks in the ECG signal. When comparing the different methods for highlighting/extracting the QRS complex (group of the three signal peaks, which is caused by the depolarization of the heart. For further information, we refer to^33^), the following method has found to be the most effective and was, therefore, used in the present study. Therefore, in a first step, a maximal overlap discrete wavelet transform (MODWT) was was used to split the ECG signal or its components into different frequency bands and thus generate its sparse representation, in which the R peaks in particular are highlighted (cf. Fig. 3a). These were then determined by using the MATLAB function “findpeaks” (cf. Fig. 3b), which is essentially based on the method presented by MathWorks for R peak detection in ECG (cf. e.g.,^42,43^). Figure 3c visualizes the resulting HRV as the underlying basis for further calculating features in the time and frequency domain.

Following the initial R peak determination, a possible effect of motion artifact on the QRS complex was investigated. A uniform time interval was determined for each R peak. The successive similarity comparison of this interval with its temporal successor using cross-correlation enables the detection of strong variations that can be attributed to movement artifacts. The resulting sections were determined based on a threshold value and excluded. In the last preprocessing step, the IBIs between the R peaks was calculated, whereby the identified noisy sequences due to motion artifacts were interpolated. This procedure is only applicable if the noisy signal sections are short (a few heartbeats). Together with the time information for the individual study sequences, the features listed above were calculated. Another advantage of interpolation is the resulting equidistant signal (cf. Fig. 3c) that can be easily transformed by the fast Fourier transform (FFT) for spectrum analysis^44^. While the ECG features were extracted as described, the respiratory features were directly available in a structured table form, so that no further preprocessing steps were necessary. In the final step, the calculated features were normalized for statistical analysis.

### Statistical analysis

Statistical analysis was performed using JASP (version 0.18.3)^45^. The dataset acquired within the study consisted of eighteen subsets. Each of these recordings contains time series of data from the $2\times 3$ different experimental conditions. To represent each of the eighteen subsets by six distinct values, representing the different conditions (thus forming a total dataset of $18\times 2\times 3$ values), the following calculation was performed: for each participant, *z*-scores were calculated over the respective mean values of the $2\times 3$ experimental conditions ($z = \frac{x-\mu }{\sigma }$ with $\mu$ being the mean value of the respective time series from one condition and $\sigma$ as its respective standard deviation). This calculation is aimed at improving the comparability of the data subsets. The $18\times 2\times 3$ z-scores were used for the statistical analysis. Here, a $2\times 3$ factorial repeated measures (rm) analysis of variances (ANOVA) was calculated with auditory stressors (“single”, “dual”) and physical stressors (“0”, “1”, “2”) as within-subject factors. The assumption of data normality was checked using both Q-Q plots (quantile-quantile plots) and the Shapiro-Wilk test. If the sphericity assumption was violated, a Greenhouse-Geisser correction was performed. In case of significant interactions within the rm ANOVA, pairwise t-tests were applied (function *pairwise_t_test* from *rstatix*) using the Holm-correction to account for multiple comparisons. Because there were no significant main effects, no further post hoc tests were applied.

All statistical tests were performed under a significance level of $\alpha = 0.05$. For the rm ANOVA, effect sizes are given as partial eta squares $\eta ^2$; for the t-tests, Cohen’s *d* was used as a measure of effect size.

## Results

### Listening effort questionnaire

**Fig. 4:**
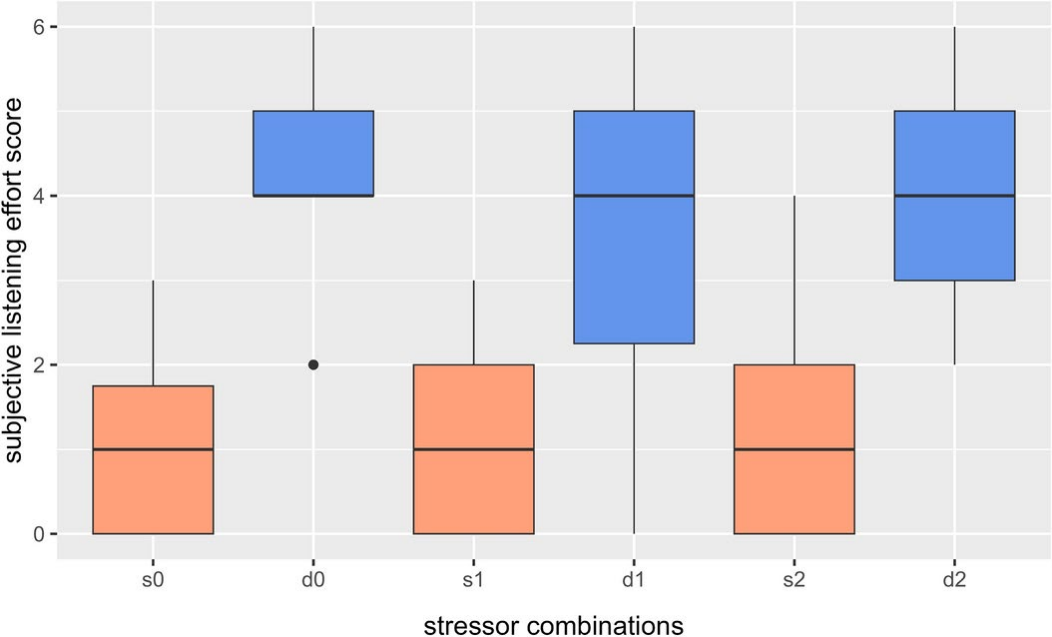
Box plots with median and quartiles for the subjective listening effort score for the single- (orange) and dual-speaker (blue) scenarios. Categorical effort scores: 0 = Effortless , 1 = very little strenuous , 2 = little strenuous, 3 = moderately strenuous, 4 = clearly strenuous, 5 = very strenuous and 6 = extremely strenuous.

Figure 4 illustrates the results of the self-rated listening effort scores. Regardless of the execution order, the situation with two speakers was perceived as more stressful. As physical stress levels increase, the scores remain approximately constant.

### Descriptive statistics

**Fig. 5:**
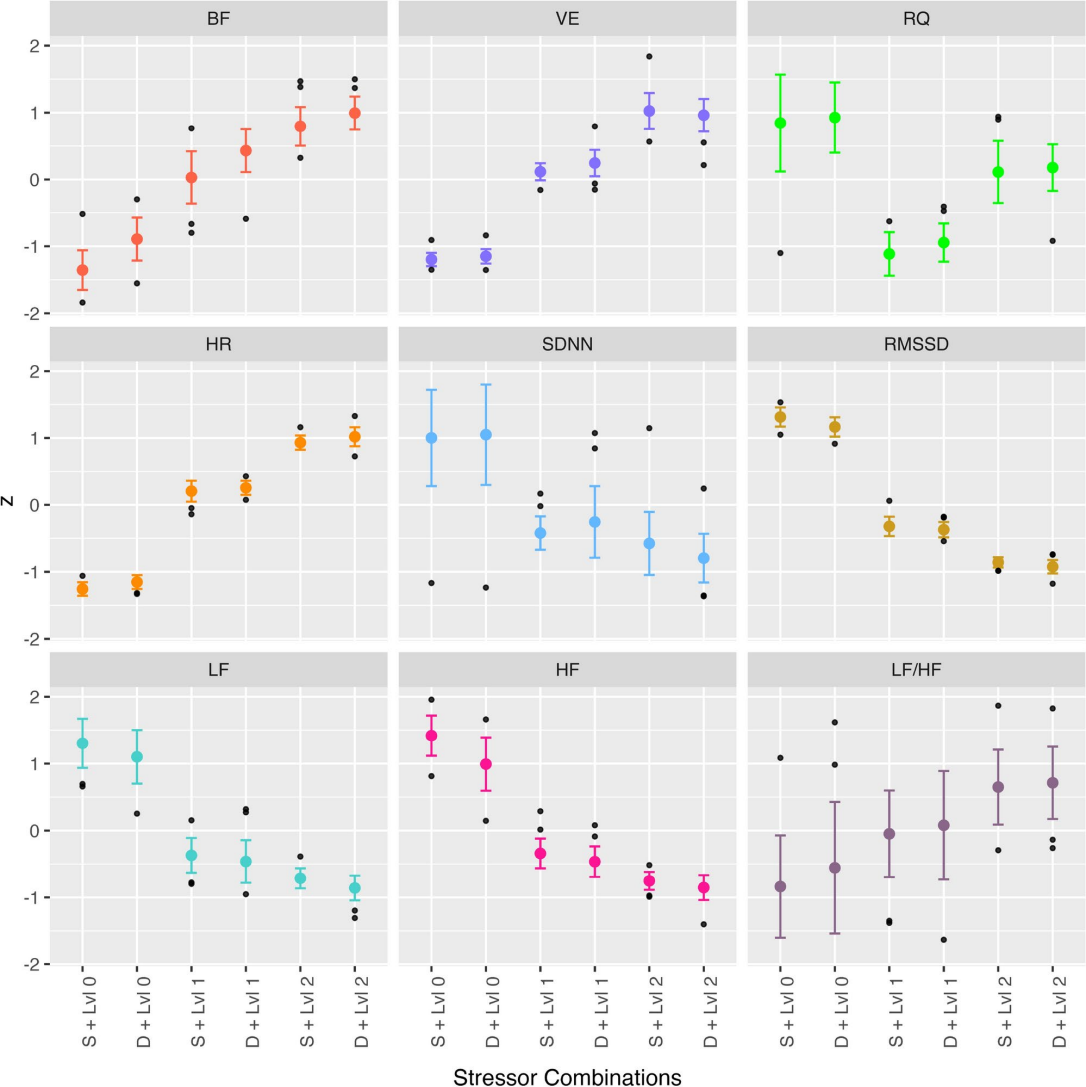
Descriptive statistics over all subject-individual *z*-values for the characteristics: Mean values with standard deviation and outliers.

**Table 1:** Mean values $(\mu )$ and standard deviations $(\sigma )$ for all level combinations the auditory (s = Single, d = Dual) and the physical (Lvl = Level, Lvl 0, Lvl 1, Lvl 2) stressors for all measured features.

	s			d		
	Lvl 0	Lvl 1	Lvl 2	Lvl 1	Lvl 2	Lvl 2
BF	$\mu = -1.356$ $\sigma = ~0.296$	$\mu = 0.029$ $\sigma = 0.393$	$\mu =0.794$ $\sigma = 0.288$	$\mu = -0.893$ $\sigma =0.321$	$\mu = ~0.432$ $\sigma = ~0.321$	$\mu = 0.994$ $\sigma = 0.47$
VE	$\mu = -1.198$ $\sigma = ~0.099$	$\mu = 0.116$ $\sigma = 0.128$	$\mu = 1.024$ $\sigma = 0.269$	$\mu = -1.150$ $\sigma = 0.108$	$\mu =~0.246$ $\sigma = ~0.198$	$\mu = 0.692$ $\sigma = 0.242$
RQ	$\mu = ~0.844$ $\sigma = ~0.724$	$\mu = -1.114$ $\sigma = ~0.325$	$\mu = ~0.111$ $\sigma = ~0.467$	$\mu = ~0.926$ $\sigma = ~0.524$	$\mu = -0.945$ $\sigma = ~0.285$	$\mu = ~0.177$ $\sigma = ~0.348$
HR	$\mu = -1.256$ $\sigma = ~0.101$	$\mu = ~0.205$ $\sigma = ~0.158$	$\mu = ~0.930$ $\sigma = ~0.106$	$\mu = -1.153$ $\sigma = ~0.104$	$\mu = ~0.256$ $\sigma = ~0.107$	$\mu = ~1.108$ $\sigma = ~0.142$
SDNN	$\mu = ~1.001$ $\sigma = ~0.721$	$\mu = -0.422$ $\sigma = ~0.249$	$\mu = -0.577$ $\sigma = ~0.470$	$\mu = ~1.049$ $\sigma = ~0.751$	$\mu = -0.255$ $\sigma = ~0.535$	$\mu = -0.796$ $\sigma = ~0.363$
RMSSD	$\mu = ~1.312$ $\sigma = ~0.143$	$\mu = -0.322$ $\sigma = ~0.145$	$\mu = -0.859$ $\sigma = ~0.076$	$\mu = ~1.165$ $\sigma = ~0.144$	$\mu = -0.371$ $\sigma =~0.144$	$\mu = -0.925$ $\sigma = ~0.100$
LF	$\mu = ~1.303$ $\sigma = ~0.367$	$\mu = -0.370$ $\sigma = ~0.260$	$\mu = -0.714$ $\sigma = ~0.150$	$\mu = ~1.101$ $\sigma = ~0.400$	$\mu = -0.462$ $\sigma = ~0.319$	$\mu = -0.859$ $\sigma = ~0.185$
HF	$\mu = ~1.418$ $\sigma = ~0.301$	$\mu = -0.341$ $\sigma = ~0.222$	$\mu = -0.752$ $\sigma = ~0.134$	$\mu = ~0.992$ $\sigma = ~0.398$	$\mu = -0.465$ $\sigma = ~0.228$	$\mu = -0.853$ $\sigma = ~0.186$
LF/HF	$\mu = -0.837$ $\sigma = ~0.765$	$\mu = -0.049$ $\sigma = ~0.646$	$\mu = ~0.649$ $\sigma = ~0.560$	$\mu = -0.556$ $\sigma = ~0.983$	$\mu = ~0.079$ $\sigma = ~0.810$	$\mu = ~0.713$ $\sigma = ~0.541$

Figure 5 and Table 1 show descriptive statistics for all analyzed respiratory and cardiac vital parameters. Of all the parameters, the differences between the single- and dual-speaker scenarios are most noticeable in BF. Differences between the three physical levels are visible in all parameters, with the exception of the ratio between the low and high frequency power components of the HRV. In this case, the data is highly scattered throughout the whole dataset. A positive trend regarding an increase in physical stress can be assumed BF, VE and HR. A negative trend can be seen for SDNN, RMSSD, LF, and HF. There seems to be a different connection for the RQ as it falls with the first increase in stimulus intensity and rises again with the second increase.

### Results of the analysis of the vital signs

First sanity checks revealed that the Zephyr ECG signal was overlaid with strong motion artifacts in the vast majority of study participants, which in some cases made it impossible to evaluate individual data sections. In addition, when putting the Zephyr belt into operation, problems due to participant-individual differences concerning the shape of the chest arose. This might also have led to the poor quality of the respective ECG signal. Fortunately, the data from the 12-lead ECG were all analyzable.

In the following, the results of the rm ANOVA are presented. The detailed results can be found in Tables 2, 3 and 4.

#### Respiratory parameters

**Table 2:** Statistical Results for the respiratory parameters: breathing frequency ($\textrm{min}^{-1}$) (BF) , respiratory minute volume ($\textrm{min}^{-1}$) (VE), and respiratory quotient (RQ) - all given as patient-individual z-scores over all six experimental conditions. The results contain values from the $2\times 3$ rm ANOVA for main effects and interactions: test statistics $F(\text {df}_{f1}, \text {df}_{f2})$, significance level *p*, and effect size (Partial Eta Squared) $\eta ^2$. In case of significant main effects, post hoc (pairwise) t-tests are reported with the test-statistic $T(\text {df}_{t})$, Holm-adjusted significance level $p_{\text {adj}}$ and effect size as Cohen’s *d*.

rm ANOVA	BF	VE	RQ
Auditory	$F(1,17) = 20.507$	$F(1,17) = 0.534$	$F(1,17) = 0.880$
	$\varvec{p < 0.001}$	$p = 0.475$	$p = 0.361$
	$\eta _p^2= 0.547$	$\eta _p^2 = 0.030$	$\eta _p^2 = 0.049$
Physical	$F(1.1, 18.74) = 496.114$	$F(1.3, 22.11) = 1840.795$	$F(1.52, 25.98) = 278.039$
	$\varvec{p < 0.001}$	$\varvec{p < 0.001}$	$\varvec{p < 0.001}$
	$\eta _p^2=0.967$	$\eta _p^2 = 0.991$	$\eta _p^2 = 0.942$
Interaction	$F(2, 34) = 1.256$	$F(1.28, 21.80) = 1.950$	$F(1.45,24.67) = 0.076$
	$p = 0.298$	$p = 0.175$	$p = 0.871$
	$\eta _p^2 = 0.069$	$\eta _p^2 = 0.103$	$\eta _p^2 = 0.004$
Post hoc			
s vs. d	$T(17) = -0.4528$		
	$$\varvec{p_\text {adj} < 0.001}$$		
	$$d = -1.129$$		
0 vs. 1	$T(17) = -20.740$	$T(17) = -37.537$	$T(17) = 23.383$
	$\varvec{p_\text {adj} < 0.001}$	$\varvec{p_\text {adj} < 0.001}$	$\varvec{p_\text {adj} < 0.001}$
	$d= -4.311$	$d= -7.278$	$d = 4.075$
0 vs. 2	$T(17) = -30.502$	$T(17) = -60.053$	$T(17) = 9.047$
	$\varvec{p_\text {adj} < 0.001}$	$\varvec{p_\text {adj} < 0.001}$	$\varvec{p_\text {adj} < 0.001}$
	$d = -6.424$	$d = -11.643$	$d = 1.576$
1 vs. 2	$T(17) = -10.162$	$T(17) = -22.516$	$T(17) = -14.336$
	$\varvec{p_\text {adj} < 0.001}$	$\varvec{p_\text {adj} < 0.001}$	$\varvec{p_\text {adj} < 0.001}$
	$d = -2.112$	$d = -4.365$	$d = 2.498$

#### BF

The results of the rm ANOVA for BF indicate significant main effects for both auditory (F(1, 17) = 20.507, $\text {p} < 0.001$) and physical stressors (F(1.1, 18.74) = 496.114, $\text {p} < 0.001$) but no interaction. Post-hoc tests revealed significant results for single vs. dual and all level comparisons of physical stress (cf. Table 2).

#### VE

The rm ANOVA for VE indicated a significant, dominant main effect for physical stress (F(1.3, 22.11) = 1840.795, $\text {p} < 0.001$). However, post-hoc tests for auditory stress did not show any significant comparisons. Auditory stress and the interaction between both stressors were not significant (cf. Table 2).

#### RQ

In the rm ANOVA the main effect for physical stress showed a significant result (F(1.52, 25.98) = 278.039, $\text {p} < 0.001$). All post-hoc comparisons showed significant effects. Neither a significant main effect for auditory stress nor an interaction between both stressors was found (cf. Table 2).

#### Cardiac parameters

**Table 3:** Statistical results for cardiac parameters of the time domain: heart rate (min${-1}$)(HR), standard deviation of the NN intervall (SDNN) and root mean square of successive differences (RMSSD). They are given as patient-individual z-scores over all six experimental conditions. The results contain values from the $2\times 3$ rm ANOVA for main effects and interactions: test statistics $F(\text {df}_{f1}, \text {df}_{f2})$, significance level *p*, and effect size (Partial Eta Squared) $\eta _p^2$. In case of significant main effects, post hoc (pairwise) t-tests are reported with the test-statistic $T(\text {df}_{t})$, Holm-adjusted significance level $p_{\text {adj}}$ and effect size as Cohen’s *d*.

rm ANOVA	HR	SDNN	RMSSD
Auditory	$F(1,17) = 4.2$	$F(1,17) = 5.6\cdot 10^{-4}$	$F(1,17) = 5.8$
	$p = 0.056$	$p = 0.981$	$\varvec{p = 0.028}$
	$\eta _p^2 = 0.198$	$\eta _p^2 = 3.3 \cdot 10^{-5}$	$\eta _p^2= 0.255$
Physical	$F(1.02,17.27) = 3137.3$	$F(1.37, 23.33)= 48.8$	$F(1.01, 17.23) = 2891.0$
	$\varvec{p < 0.001}$	$\varvec{p < 0.001}$	$\varvec{p < 0.001}$
	$\eta _p^2=0.995$	$\eta _p^2= 0.742$	$\eta _p^2= 0.994$
Interaction	$F(2,34) = 0.7$	$F(1.48, 25.13) = 1.6$	$F(1.32, 22.49)=2.0$
	$p = 0.496$	$p = 0.220$	$p = 0.171$
	$\eta _p^2 = 0.040$	$\eta _p^2=0.087$	$\eta _p^2 = 0.104$
Post hoc			
s vs. d			$T(17) = 2.411$
			$$\varvec{p_\text {adj} = 0.028}$$
			$$d= 0.709$$
0 vs. 1	$T(17) = -51.330$	$T(17) = 7.444$	$T(17) = 54.453$
	$\varvec{p_\text {adj} < 0.001}$	$\varvec{p_\text {adj} < 0.001}$	$\varvec{p_\text {adj} < 0.001}$
	$d= -11.815$	$d = 2.500$	$d = 12.841$
0 vs. 2	$T(17) = -77.914$	$T(17) = 9.343$	$T(17) =73.191$
	$\varvec{p_\text {adj} < 0.001}$	$\varvec{p_\text {adj} < 0.001}$	$\varvec{p_\text {adj} < 0.001}$
	$d = -17.934$	$d= 3.137$	$d= 17.260$
1 vs. 2	$T(17) = -26.584$	$T(17) = 1.899$	$T(17) =18.737$
	$\varvec{p_\text {adj} < 0.001}$	$p_\text {adj} = 0.066$	$\varvec{p_\text {adj} < 0.001}$
	$d= 6.119$	$d = 0.638$	$d = 4.419$

#### HR

The results of the rm ANOVA showed a statistically significant main effect for physical stress. Post-hoc tests showed significant results for all comparisons (all $p <.001$); cf. Table 3). However, neither the main effect for the auditory stressor, nor the interaction of audio and physical stress was significant (cf. Table 3).

#### SDNN

The rm ANOVA of SDNN revealed a significant main effect for physical stress only, no significant interaction or main effect for auditory stress (cf. Table 3). Post-hoc tests showed significant results for all comparisons between physical stress levels.

#### RMSSD

The rm ANOVA estimates significant main effects for both stressors, but not for their interaction (cf. Table 3). The t-test between the auditory stress levels was significant, as well as all pairwise t-tests between the physical stress levels (all $p <.05$).

#### LF

In the rm ANOVA, both stressors showed a significant main effect, but no significant interaction. The post-hoc test for the auditory stressor were significant. For the physical stressor, all post-hoc comparisons were significant.

#### HF

In the rm ANOVA, there were significant main effects for both factors, but not for their interaction. The t-test between both auditory conditions was significant, as well as all pairwise comparisons between the physical stress levels.

#### LF/HF

The rm ANOVA resulted in a main effect for physical stress but both the main effect for auditory stress and the interaction were not significant. Post-hoc tests showed for all levels of physical stress significant results.

**Table 4:** Statistical results for the cardiac features in the frequency domain: Power of the low-frequency range (0.04 Hz to 0.15 Hz) LF, power of the high-frequency range (0.15 Hz to 0.4 Hz) HF, and the ratio LF/HF - all given as patient-individual z-scores over all six experimental conditions. The results contain values from the $2\times 3$ rm ANOVA for main effects and interactions: test statistics $F(\text {df}_{f1}, \text {df}_{f2})$, significance level *p*, and effect size (Partial Eta Squared) $\eta _p^2$. In case of significant main effects, post hoc (pairwise) t-tests are reported with the test-statistic $T(\text {df}_{t})$, Holm-adjusted significance level $p_{\text {adj}}$ and effect size as Cohen’s *d*.

rm ANOVA	LF	HF	LF/HF
Auditory	$F(1,17) = 4.804$	$F(1,17) = 11.992$	$F(1, 17) = 0.854$
	$\varvec{p = 0.043}$	$\varvec{p = 0.003}$	$p = 0.386$
	$\eta _p^2 = 0.220$	$\eta _p^2 = 0.414$	$\eta _p^2 = 0.048$
Physical	$F(1.25,21.17) = 1199.968$	$F(1.26, 21.43) = 777.231$	$F(1.47, 25.06)= 31.868$
	$\varvec{p < 0.001}$	$\varvec{p < 0.001}$	$\varvec{p < 0.001}$
	$\eta _p^2 = 0.986$	$\eta _p^2= 0.979$	$\eta _p^2 = 0.652$
Interaction	$F(2.00 34.00) = 0.164$	$F(1.21,21.43) = 3.089$	$F(2,34) = 0.164$
	$p = 0.850$	$p = 0.087$	$p = 0.849$
	$\eta _p^2 = 0.010$	$\eta _p^2 = 0.154$	$\eta _p^2 = 0.010$
Post hoc			
S vs. D	$T(17) = 2.192$	$T(17) = 3.463$	
	$\varvec{p_\text {adj} = 0.043}$	$\varvec{p_\text {adj} = 0.003}$	
	$d= 0.497$	$d= 0.836$	
0 vs. 1	$T(17) = 37.484$	$T(17) = 29.831$	$T(17) = -4.125$
	$\varvec{p_\text {adj} < 0.001}$	$\varvec{p_\text {adj} < 0.001}$	$\varvec{p_\text {adj} = 0.002}$
	$d = 5.495$	$d= 6.210$	$d = -0.970$
0 vs. 2	$T(17) = 46.058$	$T(17) = 37.241$	$T(17) = -7.982$
	$\varvec{p_\text {adj} < 0.001}$	$\varvec{p_\text {adj} < 0.001}$	$\varvec{p_\text {adj} < 0.001}$
	$d = 6.752$	$d = 7.752$	$d = -1.878$
1 vs. 2	$T(17) = 8.574$	$T(17) = 7.411$	$T(17) = -3.857$
	$\varvec{p_\text {adj} < 0.001}$	$\varvec{p_\text {adj} < 0.001}$	$\varvec{p_\text {adj} < 0.001}$
	$d = 1.257$	$d = 1.543$	$d = -0.907$

## Discussion

Ongoing (over-) stimulation have a fatigue potential and are, therefore, of particular interest in the context of occupational medicine to minimize health risks, especially in hazardous work areas. With our study on healthy participants, we statistically evaluated cardio-respiratory vital features regarding the hypothesis that in the presence of both auditory and physical multi-level stressors, effects of both can be detected. To avoid a dominant reaction to a stressor, which could be caused by excessive stimulation, for example, moderate stress levels were selected.

The results of the statistical analysis showed a significant main effect for all measured parameters for the physical stressor. The auditory stressor shows significant effects for the BF, as well as for the RMSSD and both power components of the HRV spectrum. Furthermore, it was shown that there was no interaction between the effects of the two stimuli.

### Questionnaires

With regard to effective stimulus presentation, the participants statements in the questionnaires together with the use of the 6MWD justify our choice of experimental paradigm: as seen in Fig. 4, the dual-speaker scenario did indeed apply a form of auditory stress onto the participants. With the use of the 6MWD, we presume an effective provision of physical stress. During the measurement, the auditory stimulus’ sound level was adjusted to counteract the one of the treadmill. Nonetheless, solely for physical stress levels 1 and 2, some kind of background noise resulting from the treadmill operation was present. These two physical stress levels might, therefore, also contain a (small) share of auditory stimulus. Still, as most types of physical exercise also are accompanied by auditory byproducts, we find that our results remain presentable all the same.

### Respiratory parameters

The physiological changes of the cardio-respiratory system due to auditory stress occur almost instantaneously (often within milliseconds to seconds) but are often less intense compared to those induced by physical stress^46^, where significant changes are observed within minutes^47^. Petrizzo et al. also showed that reaction times to auditory stress are delayed in the presence of physical stress^48^. For the auditory stressor, our statistical analysis indicated significant main effects only for BF, RMSSD, LF, and HF. Since auditory stress has been shown to be related to a change in vital parameters and with an intensification with sustained stimulus duration^5^, a reason for this might be that the duration of the stimulus (10 min) was too short or there was a lack in stimulus amplitude for the remaining parameters to show any effect, especially for the period immediately after the start. Tipton et al. were able to show that the intensification of stress has an increasing effect on the respiratory parameters VE and BF, regardless of the type of stress^20^, which is consistent with our results on the BF.

**Fig. 6:**
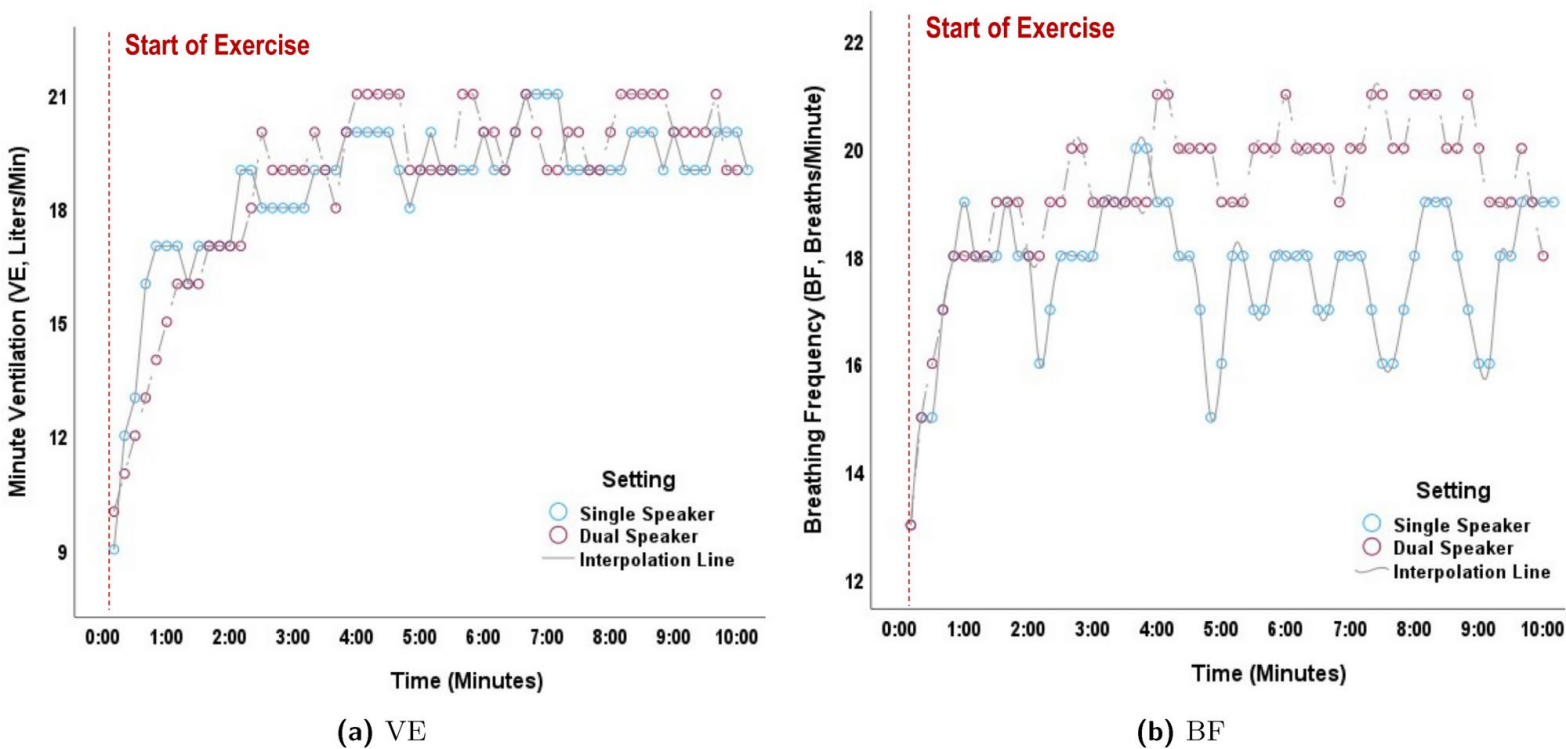
Individual example of differences in breathing pattern depending on the audiological setting. Subject exercising on a treadmill at the speed of 4 km/h and an incline of 1.5%. Although minute ventilation (VE) remains constant in the single- vs. dual-speaker setting at this level of exercise, breathing frequency (BF) is markedly elevated in the dual-speaker setting throughout the exercise phase. VE (**a**) and BF (**b**) displayed as 10-second-averaged data.

Of note, BF is higher for the dual-speaker case, while VE drops slightly. VE is a composite respiratory parameter that is calculated by BF $\times$ VT. It is known that VE is controlled within narrow limits during physical exercise by hydrogen ion concentrations, respectively CO_2_ levels, at the level of chemoreceptors^21^. A physiological increase in VE results in a raise in VT at lower exercise intensity, and an increase in BF mainly at higher exercise intensity. The differential regulation mechanisms of VT and BF are still not well understood. In our study, as expected, VE was very similar for each individual physical stress level, comparing the single-speaker vs. dual-speaker setting. However, it was surprising to see a relevant alteration in the breathing pattern at each individual physical stress level, depending on the auditory stress level (cf. Fig. 6). With higher auditory stress level (dual-speaker setting), BF was significantly increased, resulting in a more shallow and rapid breathing pattern. This may point to a selective effect of auditory stress on BF regulation, while preserving the VE set point for each physical stress level.

We consider a significant effect of the auditory stressor on the breathing pattern, especially on the changes in breathing frequency, as main result of this study as the effects were most clearly visible here (cf. Fig. 5 - BF, red) in comparison to RMSSD, LF and HF. The data for the high frequency component’s power of the HRV and the RMSSD might be significant, but its decreasing course behaves differently than research and literature shows. Further research is needed to explain this result.

### Cardiac parameters

As described in the ECG systems and cardiac parameters section, the HRV measures are related to heart-brain interactions through a reaction of the ANS that has already been studied. In the following, the results of the descriptive statistics (cf. Fig. 5) are evaluated, with the main focus set upon the cardiac vital parameter changes under increasing physical stress:

- SDNN: The negative trend with increasing exposure to stressors corresponds to the decrease in PNS activity associated with the SDNN. There appears to be a clear difference between conditions with (levels 1, 2) and without (level 0) physical stress (cf. Fig. 5, SDNN in light blue). This difference could identify the connection with the activity of the PNS.
- RMSSD: The interpretation of the negative trend of the RMSSD (cf. Fig. 5, SDNN in ochre), which is accompanied with a decrease in PNS activity, is very similar to the SDNN. However, the supposedly greater influence of PNS activity^17^ cannot be seen in our results. However, our data courses support the finding that RMSSD correlates with HF power^49^.
- Power of the low-frequency range (LF): Acharya et al. have reported that an increase in stress is associated with an increase in the power of the LF^39^. This trend is not reflected in our results.
- Power of the high-frequency range (HF): Consistent with the response described in the literature, both the PNS activity and HF power decrease according to the presence of stressors^17^.
- Ratio of LF/HF: Shows the relationship between the sympathetic (LF) and the parasympathetic stimulus (HF). Despite the unexpected decrease in the LF power with increasing stressors, the increase in the LF/HF ratio can be explained by the proportionally greater decrease in HF compared to LF.

When interpreting LF, HF and the ratio LF/HF and the associated activity of the SNS, it must be taken into account that their behavior has been validated as an indicator of controlled resting conditions. Arai et al. found that LF and HF decreased rapidly and progressively during physical activity, which is also seen in our results^50^. In addition, Perini and Veicsteinas found that during physical exercise, the HF remains present across all intensities and dominates at maximal effort, while the LF component remains stable at low intensity but significantly decreases at medium to high intensity, despite increased sympathetic activity. Factors such as fitness level, age, hypoxia, and blood distribution do not significantly affect LF power^51^. The presence of physical activity seems to have a dominant effect on regulatory mechanisms.

### Lack of auditory-physiological interaction

Throughout the entire statistical analysis, the effect sizes for the physical stressor (which is significant for all measured parameters) are always higher than the ones for the auditory conditions. However, for none of the analyzed parameters, an interaction between the auditory and physically stressing factors could be detected. This contradicts the results of Roth et al. who were able to show an additive effect of mental stress by performing digits backward problems on cardiovascular responses^52^. As mentioned above, possible reasons could lie in the intensity of the stressor on the one hand and the duration of the stimulation phases on the other. For example, a multi-speaker scenario over a longer period of time could possibly have a stronger effect. However, the challenge then lies in choosing the pauses between the study sequences long enough for the participants to have recovered to a comparable baseline state. In our study design, the order of the physical exercise levels was not randomly varied. The results show that the stress reactions between stress levels 1 and 2 are close to each other. In order to rule out order effects, the participants could repeat the scenarios several times with a random sequence of stress intensity. In addition, Huang et al. were able to show that physical fitness has a protective effect on the influence of mental stress^53^. Another influencing factor is sensory implication, which refers to the effects and significance of sensory experiences on behavior, perception and cognition. In this study, habituation effects (in particular auto-regulatory mechanisms) or previous experiences with auditory stress were not taken into account. Further research on the interaction mechanisms is necessary to investigate the effects on the respiratory parameters in particular and the spectral HRV parameters and their dependence on physical activity in more detail.

Nevertheless, all measured parameters with a significant main effect of the auditory stressor can be extracted directly from the ECG signal. There are methods of extracting information on the respiratory cycle indirectly via ECG-derived respiration (EDR)^54–56^. This is promising in view of how both types of stress can possibly be extracted from ECG data. Taking this finding in mind, continuous monitoring of worker’s vital parameters can help identifying sources of stress. This opens up the possibility of finding strategies to increase resilience on the one hand and to minimize individual stress on the other, e.g. by taking breaks. Especially in an occupational context, the quality of people’s work can depend on their physical and mental state. Wearable sensors for vital signs monitoring show promising potential for a growing field of applications. There are several measurement points for different sensor types. The human ear is particularly interesting as a perspective example. Here, modern hearing systems can be expanded to include non-invasive sensors for temperature, acceleration and movement^57^, oxygen saturation, heart rate and HRV, as well as EEG sensors for measuring brain activity^58^. With this, in the field of occupational health, hearing aid wearers in particular could have the opportunity to identify potential risks of overload in order to initiate suitable countermeasures.

## Conclusion

Our work focused on the effects of auditory and physical stressors on cardio-respiratory vital signs. For the analysis of cardio-respiratory stress response, a prospective, pseudo-randomized single-center study designed on young, healthy subjects was conducted. The hypothesis pursued was that in the presence of both auditory and physical multi-level stressors, effects of both can be detected in the analysis of cardio-respiratory vital features. Furthermore, we assumed an increasing effect with rising stimuli intensity together with an additive interaction.

The study concludes that both physical and auditory stressors elicit measurable effects on cardio-respiratory parameters, with physical stressors exerting more pronounced effects across all measured variables compared to auditory stressors. A key finding is that auditory stress significantly alters BF, leading to a shallower breathing pattern while maintaining constant VE, indicating a selective regulatory effect of auditory stress on respiratory dynamics without compromising overall ventilation. Contrary to previous research suggesting additive effects of combined stressors, the present study found no interaction between physical and auditory stress. This deviation from earlier findings may be attributable to the intensity and duration of the stimuli. In particular, no significant interaction effects were observed, which contrasts with prior studies that reported cumulative impacts of stressors on cardiovascular responses. Furthermore, HRV parameters, including RMSSD and HF, exhibited significant trends; however, their responses diverged from established literature, suggesting the need for further inquiry to clarify these discrepancies.

In summary, the study indicates that auditory and physical stressors operate independently, with breathing frequency emerging as the most sensitive indicator of auditory stress. Future research should focus on the impact of stressor amplitude and duration, as well as potential physiological interactions, to better understand the mechanisms underlying these responses.

The results affirm that stress-related parameters, including respiratory information via EDR, can be reliably extracted from ECG data. Continuous monitoring of workers’ vital signs using wearable sensors, particularly integrated into hearing aids, holds great potential in occupational health. Such systems could help detect signs of overload, allowing for early intervention to mitigate stress and improve work performance.

## Data Availability

The datasets generated and/or analysed during the current study are not publicly available due to the restrictions of the General Data Protection Regulation of the European Union. However, researchers who are interested in the data should contact the corresponding author. Then, in a bilateral process, a solution for the data exchange can be found in compliance with legal and ethical restrictions.
